# Edge Preserved Speckle Noise Reduction Using Integrated Fuzzy Filters

**DOI:** 10.1155/2014/876434

**Published:** 2014-10-30

**Authors:** Nagashettappa Biradar, M. L. Dewal, Manoj Kumar Rohit

**Affiliations:** ^1^Indian Institute of Technology Roorkee, Roorkee 247 667, India; ^2^Post Graduate Institute of Medical Education and Research, Chandigarh 160 012, India

## Abstract

Echocardiographic images are inherent with speckle noise which makes visual reading and analysis quite difficult. The multiplicative speckle noise masks finer details, necessary for diagnosis of abnormalities. A novel speckle reduction technique based on integration of geometric, wiener, and fuzzy filters is proposed and analyzed in this paper. The denoising applications of fuzzy filters are studied and analyzed along with 26 denoising techniques. It is observed that geometric filter retains noise and, to address this issue, wiener filter is embedded into the geometric filter during iteration process. The performance of geometric-wiener filter is further enhanced using fuzzy filters and the proposed despeckling techniques are called integrated fuzzy filters. Fuzzy filters based on moving average and median value are employed in the integrated fuzzy filters. The performances of integrated fuzzy filters are tested on echocardiographic images and synthetic images in terms of image quality metrics. It is observed that the performance parameters are highest in case of integrated fuzzy filters in comparison to fuzzy and geometric-fuzzy filters. The clinical validation reveals that the output images obtained using geometric-wiener, integrated fuzzy, nonlocal means, and details preserving anisotropic diffusion filters are acceptable. The necessary finer details are retained in the denoised echocardiographic images.

## 1. Introduction

Coherent imaging modalities such as synthetic aperture RADAR (SAR), optical coherence tomography (OCT), and ultrasound (US) suffer due to the presence of speckle noise [1–4]. Multiplicative nature of speckle noise complicates denoising process as it would be necessary to remove noise with edges and structure being well preserved [4–6]. Various types of filters such as anisotropic diffusion (AD) [7–10], wavelets [11–16], m-band ridgelet [17, 18], adaptive [19, 20], total variation [21, 22], bilateral [23], nonlocal means (NLM) [24, 25] and Ripplet [26, 27] are used for reduction of speckle noise. The performance of each filter depends on the type of noise and image.

Basic noise reduction techniques such as median, adaptive weighted median (AWM), and moving average (MAV) filters are known for suppression of additive noise but their application on ultrasound images is less researched [1]. The function of AWM filter depends on the size of the window along with weight adjustment [1, 3, 5]. It operates using a fixed window size restricting the enhancement phenomenon and often results in smeared boundaries. Geometric filter is a nonlinear, iterative filter which takes into account the image morphology. It tries to match the similarity of centre pixel in window to the values in the neighborhood [6]. It is known for edge preservation and noise reduction in ultrasound images. But the issue of concern is that some amount of noise is retained in geometric filtered image. The wiener filter uses the minimum mean square error (MSE) constraint for estimating a noise free image. Adaptive wiener filter is employed in additive noise reduction applications. It is used for speckle noise reduction in logarithmic domain and has become reference noise reduction for many researchers to compare results obtained by their respective methods or modifications [12, 15]. According to psychophysics, human eye does not function as minimum MSE estimator; in fact, it allows noise leading to sharp intensity changes.

Noise reduction capability of diffusion based despeckling is up to mark when noise contamination is higher, as in the case of OCT [3] images. In NLM based denoising, patches around a pixel are considered instead of comparing the intensities of the pixels [24]. An extension of NLM filter was proposed by Deledalle et al. [25] by incorporating noise distribution model instead of computing Euclidean distance for pixel similarity calculations. Experimentations in [25] were performed by embedding synthetic additive and multiplicative noise in standard test images such as Barbara, Lena, Boat, and House. The results are reported in terms of SNR in [25]. Recently, Mateo and Fernández-Caballero [1] tested median filter with different window sizes on ultrasound image of kidney and it was concluded that, with increase of window size, the boundaries become hazier and more details disappear. It is also stated in [1] that edge preservation index decreases with an increase in window size. The value edge preservation index was very small (<0.1). Poor noise removing capability, loss of finer details, and selection of appropriate window size and shape are the basic issues which need to be sorted out in basic techniques [1, 3].

Fuzzy filters with membership functions defined in terms of moving average and median center were tested and proven to be effective in reducing various types of additive noise [28, 29]. Also, the performances of these filters, for additive noise reduction, are reported and analyzed using MSE as reference metrics of evaluation. The despeckling application of fuzzy filters is not extensively analyzed and reported for ultrasound images. Therefore, this paper studies the applications of fuzzy filters along with geometric, wiener, combination of geometric-wiener, combination of geometric-fuzzy, and the proposed integrated fuzzy (i.e., geometric-wiener-fuzzy) filter. Traditional parameters like peak signal to noise ratio (PSNR), mean square error (MSE), and signal to noise ratio (SNR) do not speak of edge preservation and hence are not sufficient for evaluating despeckling filter applications in ultrasound images [1]. Hence, it is proposed to evaluate and analyze the despeckling characteristics in terms of 16 image quality metrics (IQM). Edge and structural preservation are evaluated using beta metric (*β*), figure of merit (FoM), structural similarity (SSIM), and image quality index (IQI) in addition to traditional parameters such as SNR, PSNR, MSE, and correlation coefficient (*ρ*).

## 2. Denoising Techniques

A novel speckle reduction technique based on integration of geometric, wiener, and fuzzy filters is proposed and analyzed in terms of noise suppression and edge preservation capabilities. The fuzzy filters based on triangular function with median (TMED) center, asymmetrical triangular function with median (ATMED), moving average (ATMAV) center, and symmetrical triangular moving average (TMAV) center are analyzed for multiplicative speckle noise reduction applications.

### 2.1. Modeling Employed for Denoising

The multiplicative speckle noise is modeled as

(1) $$\begin{array}{c} f\left(i,j\right)=g\left(i,j\right)n\left(i,j\right), \end{array}$$

where *g*(*i*, *j*) is noise free image, *f*(*i*, *j*) is the acquired image, *n*(*i*, *j*) is the multiplicative noise, and *i* and *j* are the variables indicating the spatial locations [1]. The process of converting multiplicative noise into approximated additive noise is performed by projecting image into logarithmic space [1] as follows:

(2) log⁡⁡fi,j=log⁡⁡gi,jni,j=log⁡gi,j+log⁡ni,j.

The above equation (2) is rewritten with *f*_*ij*_ = log⁡[*f*(*i*, *j*)], *g*_*ij*_ = log⁡[*g*(*i*, *j*)], and *n*_*ij*_ = log⁡[*n*(*i*, *j*)] as

(3) $$\begin{array}{c} f_{ij}=g_{ij}+n_{ij}. \end{array}$$

Equation (3) makes way for application of methods developed for additive white Gaussian noise to be tested and analyzed on images containing multiplicative speckle noise. In these methods, the input is projected into logarithmic space, *f*(*i*, *j*) = log⁡[*f*(*i*, *j*)] and output is obtained by taking exponential of the denoised image as represented in (4)

(4) $$\begin{array}{c} \hat{g}\left(i,j\right)=\exp\left(\text{Method}\left(\log\left(f\left(i,j\right)\right)\right)\right), \end{array}$$

where “Method” represents filter used.

### 2.2. Geometric Filter

The geometric filter works by increasing or decreasing the pixel values in the neighborhood based on their comparative values [6]. The intensity of the pixel located at the center of 3 × 3 window is compared with eight neighbors. Depending on the intensity values of neighborhood pixels, the value is either incremented or decreased so that the values stand out compared to others. The size of moving window in this study is set to 3 × 3 with number of iteration being equal to 2. The visual quality of the image improves on using geometric filter but at the same time the image is smoothed considerably also with some noisy edge retained. Some of the edges and finer details are mostly lost.

### 2.3. Fuzzy Filters

Median filter effectively suppresses the speckle noise but the edges are not well preserved [28, 29]. Fuzzy filters with median center preserve image sharpness, when used for additive noise reduction. The median value based on fuzzy triangulation membership function with median center (TMED) [28, 29] is defined by (5) with different window and padding size

(5) Ffi+r,j+s =1−f(i+r,j+s)−fmed(i,j)fmm(i,j),iiiiiifor  fi+r,j+s−fmedi,j≤fmmi,j,1,iiifor  fmm=o,fmmi,j=max⁡⁡fmax⁡i,j−fmedi,j,iiiiiiiiiiiiiiiiiiiiiiiiiiifmedi,j−fmin⁡i,j.

The maximum, minimum, median, and moving average values are, respectively, represented by *f*_max⁡_(*i*, *j*), *f*_min⁡_(*i*, *j*), *f*_med_(*i*, *j*), and *f*_mav_(*i*, *j*) with *s*, *r* ∈ *A*, the window at indices (*i*, *j*).

The output of the fuzzy filters is estimated using (6), given below as follows:

(6) $$\begin{array}{c} y\left(i,j\right)=\frac{\sum_{(r,s)\in A}F\left[f\left(i+r,j+s\right)\right]\cdot f\left(i+r,j+s\right)}{\sum_{(r,s)\in A}F\left[f\left(i+r,j+s\right)\right]}, \end{array}$$

where *F*[*f*(*i*, *j*)] are the window function defined in terms of fuzzy membership functions and “*A*” is area. The median filtering using fuzzy asymmetrical triangulation membership function with median center (ATMED) is defined by (7) [28, 29] below as

(7) Ffi+r,j+s =1−fmedi,j−fi+r,j+sfmedi,j−fmin⁡i,j,iiiiiifor  fmin⁡i,j≤fi+r,j+s≤fmedi,j,1−fi+r,j+s−fmedi,jfmax⁡i,j−fmedi,j,iiiiiifor  fmedi,j≤fi+r,j+s≤fmax⁡i,j,1,iiifor  fmedi,j−fmin⁡i,j=0iiiiiior  fmax⁡i,j−fmedi,j=0.

The details of moving average filter using fuzzy triangulation membership function (TMAV) with moving average center and moving average using asymmetrical fuzzy triangulation membership function (ATMAV) are available in [28, 29].

## 3. Evaluation of Denoising Techniques

The applications of denoising filters are analyzed on both echocardiographic and standard test images. Echocardiographic images are inherently noisy and standard test images are artificially embedded with speckle noise. The despeckling filters are evaluated by using IQM and visual quality assessment. The performance estimation in standard and echocardiographic images is different; in the standard test images, performance is computed relative to noise free reference image, whereas, in clinical images, the measurements are with respect to the original speckled input. The standard image is artificially embedded with speckle noise using MATLAB inbuilt function imnoise with different values of noise variance.

### 3.1. Echocardiographic Image Database

The denoising filters are applied to the database consisting of echocardiographic images. The TTE images are acquired during diastole and systole using different parasternal and apical windows. Echocardiographic image database is completely anonymized, with patient information provided. The approval of institutional ethics committee is obtained for the usage of clinical echocardiographic image database for preprocessing and analysis.

### 3.2. Image Quality Metrics

The denoising capabilities of GF, WF, GWF, F1 to F4, GF1 to GF4, and GWF1 to GWF4 filters are evaluated using PSNR, MSE, *ρ*, and SNR using original image *f*_org_ and denoised image *f*_den_ [1, 2]; edge preservation and distortion are measured using FOM, *β*, and SSIM [2, 4, 30]. The parameters such as normalized mean square error (NMSE), root mean square error (RMSE), normalized error summation (Err3, Err4), and geometric average error (GAE) are also being employed in the analysis [30]. Some of the parameters such as PSNR [1, 30], MSE [1, 2], *β* [1], and IQI [30] are defined below as

(8) PSNRfden,forg=20×log⁡10255MSEfden,forg,MSEfden,forg=1MN∑i=1M∑j=1Nfden−forg2,SNR=10×log⁡10varforgMSE(fden,forg),FoMfden,fref=1max⁡(Nden,Nref)∑j=1Nden11+γdj2,ρfden,forg=∑i=1M∑j=1Nfden·forg∑i=1M∑j=1Nfden2∑i=1M∑j=1Nforg2,β=DΔfden−Δ−forg,Δforg−Δ−forg×DΔfden−Δ−fden,Δfden−Δ−fdeniiiiiii·DΔforg−Δ−forg,Δforg−Δ−forg−1/2,SSIMforg,fden =1M∑2μforgμfden+c12σforgfden+c2μforg2+μfden2+c1σforg2+σfden2+c2,

where *γ* is the scalar multiplier being utilized as penalization factor with typical value 1/9, *n*_*d*_ and *n*_*r*_ are the number of pixels in original and processed images, respectively, *d*_*j*_ is the Euclidean distance, Δ*f*_den_ and Δ*f*_org_ represent the filtered version of original and processed images, pixel mean intensities in the region Δ*f*_den_, Δ*f* are represented by $\overset{-}{\Delta }f_{\text{den}}$ and $\overset{-}{\Delta }f_{\text{org}}$, respectively, and *c*_1_ and *c*_2_ are constants.

## 4. Proposed Integrated Fuzzy Filters

**Figure 1:**
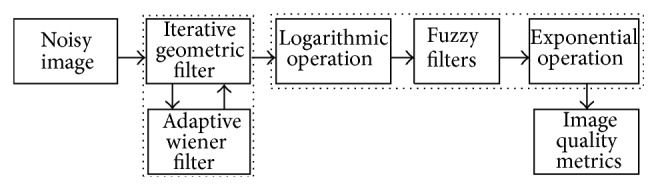
Block diagram of the proposed integrated fuzzy filter.

The noise suppression capabilities of the fuzzy filters are within acceptable limits in logarithmic domain but the edges are not preserved. The iterative geometric filter is known for edge preservation when employed for speckle noise reduction but retains noisy edges in the denoised image. Therefore, it is proposed to integrate the edge preservation capabilities of geometric filter and noise reduction capabilities of wiener filter into fuzzy filters. During the implementation of fuzzy filters such as TMED, ATMED, TMAV, and ATMAV, as shown in Figure 1, geometric-wiener filtered image is logarithmically transformed; fuzzy membership function with either median or moving average centre is calculated symmetrically or asymmetrically.

The output of fuzzy filter is transformed to the nonlogarithmic space using exponential operation.

Initially, experiments are conducted to study the applications of geometric filter (GF), wiener filter (WF), and combination of geometric and wiener (GW) filter, considering the multiplicative noise model. Analysis of GF, WF, and GW filters is followed by the study of fuzzy filters in the logarithmic domain, considering approximated additive noise model. The fuzzy filters based on TMED, ATMED, TMAV, and ATMAV are numbered as F1, F2, F3, and F4, respectively. The sequential combinations of geometric filter and fuzzy filter are numbered as GF1, GF2, GF3, and GF4. The integration of geometric-wiener with fuzzy filters, known as integrated fuzzy filters, is represented as GWF1, GWF2, GWF3, and GWF4, where F1 to F4 are fuzzy filters and W represents wiener filter.

The geometric filter is used for denoising of echocardiographic images with different number of iteration and the number of iteration is taken as two in the proposed integrated fuzzy filters. The wiener filter is used at the end of each iterative loop during the integration of geometric filter with wiener filter. Geometric filter is embedded with fuzzy filters in the second set of experiments. Finally, geometric-wiener is combined with fuzzy filters as shown in Figure 1 and the steps are explained below.

The steps incorporated for implementation of integrated fuzzy filters are as follows.

**Step 1 .**

Consider standard noise free image, resize the image to 512 × 512, convert it to gray scale, and embed synthetic speckle noise using imnoise with different variance. In case of echocardiographic images, no artificial noise is added as they are inherent with speckle noise.

**Step 2 .**

The images are denoised using iterative geometric-wiener filter with different number of iterations. During iterative process, the geometric filtered image is subjected to adaptive wiener filtering.

**Step 3 .**

Project the denoised image into the logarithmic space according to (2). The output is of the form *f* = log⁡(double(*f*) + 1), where *f* is output image of geometric filter.

**Step 4 .**

Image in the logarithmic domain is filtered using fuzzy filter. The output of the fuzzy filter is estimated using (6).

**Step 5 .**

The output of fuzzy filter is projected back to the nonlogarithmic space using exponential operation which is of the form *Y*_denoised_ = exp⁡(*y*) − 1.

**Step 6 .**

The image quality metrics are computed followed by the clinical validation of echocardiographic images in terms of enhancement and details retention.

The MATLAB inbuilt “wiener2” is being used for implementation of wiener filtering. The window size of wiener filter is taken as 3 × 3. The above steps are being repeated for different levels of noise artificially added onto the noise free images and for different window size of fuzzy and wiener filters varying in the ranges 3 × 3, 5 × 5, 7 × 7, and 9 × 9. Further, all the steps are repeated using TMED, ATMED, TMAV, and ATMAV filters. In each experiment, Step 4 is different, as each is based on one of the four fuzzy filters. All experimentations are performed using several standard test images such as Lena, Mandrill, Cameraman, Barbara, Monarch, woman dark hair, and House of size 512 × 512 [25]. The applications of geometric, wiener, combination of geometric-wiener, combination of geometric-fuzzy, and integrated fuzzy filters are analyzed and studied on echocardiographic images also.

## 5. Results

The performance of geometric, wiener, geometric-wiener, fuzzy, geometric-fuzzy, and geometric-wiener-fuzzy filters is analyzed in terms of IQM followed by visual assessment. Performance of these filters at various noise levels is tabulated in Table 1. The results in Table 1 reveal that the performance of GF filters is superior compared to fuzzy filters. These results are further improved in case of proposed integrated fuzzy filters at all noise levels in comparison to results of fuzzy and geometric-fuzzy filters. FoM, SSIM, IQI, *β*, *ρ*, SNR, and PSNR values are highest in case of GWF filters in comparison to fuzzy and geometric-fuzzy filters. The SNR and PSNR are higher by 2 dB, for GWF2 compared to GF2 filter at all noise levels.

The following are the highlights of the results tabulated in Table 1: (1) the performance of F3, GF3, and GWF3 filters is superior in terms of edge preservation compared to filtering based on F1 and F2. The values of FoM, SSIM, IQI, and *β* are higher for these filters suggesting better edge preservation. (2) The noise reducing capabilities of F2 based filters are better compared to filters based on F1 and F2. The higher values of SNR and PSNR with lesser MSE reveal this point suggesting better noise reduction using F2, GF2, and GWF2.

(3) Improvement in image quality metrics is observed using the integration of geometric with fuzzy and geometric-wiener-fuzzy filters. Enhancement is observed in GF filters compared to fuzzy filters and GWF filters when compared to GF filters. The results suggest that proposed methods are superior compared to others.

Result analysis based on Tables 2 and 3 reveals that the performance of filters is on similar lines as discussed in earlier paragraph for all images. The IQM obtained for various images at noise variance equal to 0.01 are tabulated in Table 2. The results in terms of FoM, *ρ*, IQI, SSIM, MSE, RMSE, ERR3, and ERR4 are tabulated in Table 2. Performance in terms of LMSE, *β*, SNR, and PSNR is shown in Table 3. The analyses show improvement in all parameters, for almost all images, on comparing performance of geometric-fuzzy filters with geometric-wiener-fuzzy filters. It is also observed that the performance of geometric-wiener filter is superior compared to geometric filter. The values of FoM ≥ 0.75, *ρ* ≥ 0.99, and SSIM ≥ 0.9 for GWF filters indicate that the results are within the acceptable limits in terms of edge and structure preservation. The IQI values are enhanced using GWF filters as observed in Table 2. The performance of geometric-wiener filter is best in terms of SSIM, even higher compared to all fuzzy, GF, and GWF filters. MSE is reduced by more than two times using GW and GWF filters for all images at noise variance equal to 0.01. The RMSE, LMSE, ERR3, and ERR4 are also reduced using GW and GWF filters. Beta metric values are greater than 0.7 using wiener and GWF2 filters, whereas for all other methods the value is less than 0.5. But improvement in *β* value is observed using GW, GF, and GWF filters. SNR and PSNR values tabulated in Table 3 reveal that the performance of GF1 is enhanced by more than 2 dB in the proposed GWF1 filter for all images except in case of Barbara image. Performance of GF4 is increased by more than 4 dB using proposed GWF4 filter in case of all images except for “woman dark hair” image. The overall result analysis reveals performance improvement using the proposed filters with the edges and structure being well preserved with maximum noise suppression.

**Figure 2:**
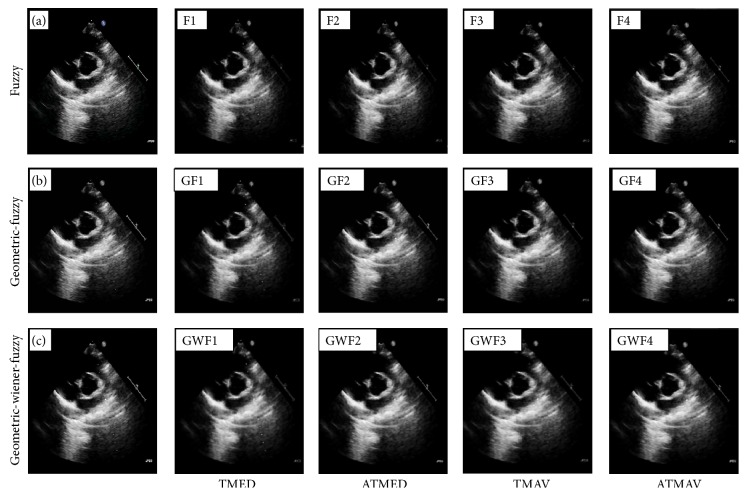
Denoised echocardiographic images: (a) original image, (b) geometric filter, (c) geometric-wiener filter, F1 to F4: TMED, ATMED, TMAV, and ATMAV, GF1 to GF4: geometric-fuzzy filters, and GWF1 to GWF4: geometric-wiener-fuzzy filters.

**Figure 3:**
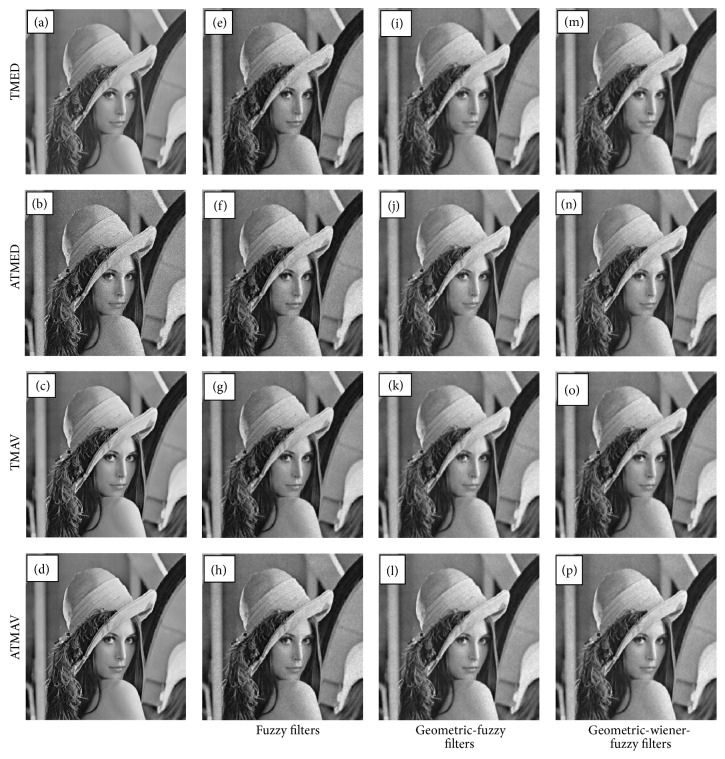
Visual quality comparison of denoised Lena image for noise level equal to 0.01: (a) original noise free image, (b) noisy image, (c) geometric filter, (d) geometric-wiener filter, (e) to (h) F1 to F4 filter, (i) to (l) GF1 to GF4 filter, and (m) to (p) GWF1 to GWF4 filter.

The visual qualities of denoised echocardiographic images and standard test image are shown in Figures 2 and 3, respectively. The visual quality of denoised images, that is, GWF filters, is compared to fuzzy filters (F1 to F4) and geometric-fuzzy (GF1 to GF4) filters. It is observed that large amount of noise is retained in fuzzy filters. Noise reduction is more pronounced using GW and GWF filters. The clinical validation of denoised images is carried out by clinical practitioners at Post Graduate Institute of Medical Education and Research, Chandigarh, India. The clinicians validated the denoised images based on details and structure preservation from medical practitioners' perspective. Denoised images based on GF, GWF, and GW filters were acceptable for the doctors, whereas the denoised images obtained using TMED (F1) and GF1 were not appreciated.

The performances of geometric-wiener (GW), geometric-fuzzy using TMAV (GF3), and geometric-wiener-fuzzy using TMED and ATMED (GWF1 and GWF2) are compared with 21 existing denoising techniques in Table 4 in addition to comparison with four fuzzy filters and wiener filter as already discussed in the above paragraphs. The MATLAB functions provided by the authors of NLM [24], PPB [25], ATV [21], Ripplet [26], and bilateral filters [23] are being used in the implementations. Parameters for all the methods were based on the results and discussion in various research papers. The parameters are selected such that each filter results in the optimum values.

The results tabulated in Table 4 are for Lena image embedded with speckle noise of variance 0.01. The value of FoM is highest for GF3 filter, suggesting that minimum distortion is being introduced in the denoised image with edges being well preserved. The performance of GWF1 and GWF2, in terms of FoM, is superior compared to FoM of all other methods except for NLM based denoising. The IQI of GW, GF3, and GWF2 is superior compared to all other methods except for NLM based denoising techniques. IQI of NLM and PPB are fractionally higher compared to GW, GF3, and GWF2. The SSIM values are greater than 0.9, indicating high structural similarity between original and denoised images. SSIM of GF3 and GWF2 are higher compared to all other methods except for PPB, NLM, and GLM based despeckling techniques. SNR of GF3 is 52.14 dB at noise variance = 0.01, which is higher compared to all other methods except for NLM and PPB based denoising. The PSNR of GWF2, ATV, GLM, and PPB are 31.73 dB, 32.33 dB, 32.15 dB, and 33.99 dB, respectively, suggesting that the performance is on similar lines except for PPB filter. Based on the result analysis, it is observed that the proposed integrated fuzzy filters enhance the despeckling performance of fuzzy and geometric filters. The performances of integrated fuzzy filters are superior compared to all methods except for NLM based despeckling filters. The clinical validation reveals that output images obtained using MPT, MBR, Ripplet, and PMAD are not acceptable and medically not useful. The performance of NLM, PPB, GWF2, GW, GWF3, GLM, and DPAD is clinically acceptable.

## 6. Conclusion

The edge preservation capabilities of fuzzy filters are enhanced with integration of geometric-wiener filter. Higher amount of speckle noise is suppressed along with the edges and structures being well preserved using the proposed integrated fuzzy filters. The performance of geometric filter is enhanced by embedding wiener filter, during the iterative processing. The noise retained in geometric filtered image is suppressed using wiener filter in the proposed despeckling methods. The despeckling applications of fuzzy filters are studied and analyzed in this paper. Denoising characteristics of fuzzy filters are improved by embedding geometric-wiener filter with fuzzy filters. FoM, SSIM, IQI, *β*, *ρ*, SNR, and PSNR values are highest in case of GWF filters in comparison to fuzzy and geometric-fuzzy filters. The SNR and PSNR are higher by 2 dB, for GWF2 compared to GF2 filter at all noise levels. SNR and PSNR values tabulated in Table 3 reveal that the performance of GF1 is enhanced by more than 2 dB in the proposed GWF1 filter for all images except in case of Barbara image. Performance of GF4 is increased by more than 4 dB using proposed GWF4 filter in case of all images except for “woman dark hair” image. The overall result analysis shows improvement in the performance of fuzzy filters and geometric filters using the proposed scheme of integration. Also, the performances of integrated fuzzy filters are superior compared to all methods except for NLM based despeckling filters. The clinical validation reveals that the performance of GW, GWF, NLM, PPB, GLM, and DPAD filters is acceptable, with required details being well preserved.

## Figures and Tables

**Table 1:** Comparison of IQM for fuzzy, geometric-fuzzy, and integrated fuzzy filters.

Noise	Method	FoM	SSIM	IQI	*β*	*ρ*	SNR	PSNR	MSE
0.01	F1	0.7514	0.8659	0.4917	0.0755	0.9966	43.25	27.28	121.56
	GF1	0.8259	0.8697	0.4947	0.0931	0.9966	43.38	27.34	119.84
	GWF1	**0.8671**	**0.8905**	**0.5431**	**0.1518**	**0.9968**	**43.82**	**27.57**	**113.81**
	F2	0.7590	0.8750	0.5530	0.3016	0.9984	49.94	30.62	56.31
	GF2	0.7932	0.8835	0.5652	0.3284	0.9985	50.57	30.94	52.35
	GWF2	**0.8819**	**0.9202**	**0.6163**	**0.4552**	**0.9988**	**52.16**	**31.73**	**43.62**
	F3	0.9066	0.9221	0.6002	0.0807	0.9982	48.62	29.97	65.51
	GF3	0.9099	0.9228	0.6002	0.0701	0.9982	48.87	30.09	63.66
	GWF3	0.8689	0.9106	0.5918	0.0675	0.9980	47.86	29.59	71.50

0.05	F1	0.4435	0.6930	0.3646	0.0080	0.9931	35.16	23.24	308.48
	GF1	0.4563	0.6993	0.3665	0.0001	0.9933	36.00	23.65	280.30
	GWF1	**0.5597**	**0.7816**	**0.4293**	0.0284	**0.9956**	**41.01**	**26.16**	**157.29**
	F2	0.4023	0.6781	0.3990	0.1315	0.9946	39.24	25.28	192.90
	GF2	0.4239	0.6845	0.4049	0.1374	0.9950	39.96	25.64	177.63
	GWF2	**0.5149**	**0.7707**	**0.4755**	**0.2384**	**0.9970**	**44.35**	**27.83**	**107.16**
	F3	0.5465	0.7950	0.4647	0.0888	0.9968	41.18	26.25	154.31
	GF3	0.5383	0.8021	0.4704	0.0878	0.9969	42.46	26.89	133.17
	GWF3	**0.5990**	**0.8032**	**0.4762**	0.0910	**0.9969**	**44.26**	**27.79**	**108.27**

0.1	F1	0.3627	0.5671	0.2869	0.0230	0.9864	27.36	19.34	757.23
	GF1	0.3659	0.5757	0.2924	0.0152	0.9869	28.34	19.83	676.50
	GWF1	**0.4419**	**0.6865**	**0.3661**	−0.0003	**0.9932**	**36.92**	**24.12**	**252.01**
	F2	0.3693	0.5691	0.3265	0.0854	0.9898	33.53	22.42	372.49
	GF2	0.3660	0.5776	0.3341	0.0860	0.9904	34.32	22.81	340.12
	GWF2	**0.4535**	**0.6728**	**0.4067**	**0.1670**	**0.9948**	**39.73**	**25.52**	**182.38**
	F3	0.4040	0.6821	0.3833	0.0764	0.9940	33.35	22.33	380.09
	GF3	0.4020	0.6927	0.3903	0.0732	0.9943	34.75	23.03	323.62
	GWF3	**0.4634**	**0.7163**	**0.4148**	**0.0868**	**0.9953**	**40.22**	**25.77**	**172.35**

0.2	F1	0.3164	0.3917	0.1851	0.0174	0.9626	17.42	14.36	2380.26
	GF1	0.3141	0.4038	0.1932	0.0176	0.9654	18.41	14.86	2124.11
	GWF1	**0.3750**	**0.5611**	**0.2952**	**0.0180**	**0.9872**	**30.73**	**21.02**	**513.91**
	F2	0.3283	0.4495	0.2518	0.0582	0.9794	27.09	19.20	781.68
	GF2	0.3294	0.4549	0.2564	0.0552	0.9802	27.79	19.55	721.01
	GWF2	**0.3798**	**0.5643**	**0.3381**	**0.1162**	**0.9906**	**34.49**	**22.90**	**333.25**
	F3	0.3399	0.5072	0.2695	0.0474	0.9833	22.78	17.05	1283.26
	GF3	0.3479	0.5172	0.2752	0.0486	0.9847	24.04	17.67	1110.79
	GWF3	**0.3845**	**0.5952**	**0.3376**	**0.0725**	**0.9912**	**34.04**	**22.67**	**351.27**

**Table 2:** Comparison of IQM for proposed filters on different images with noise variance equal to 0.01.

Methods	FoM						*ρ*				
	Lena	Darkhair	Blonde	Peppers	Mandrill	Barbara	Lena	Darkhair	Blonde	Peppers	Mandrill
GF	0.7382	0.5140	0.7329	0.6516	0.9020	0.7529	0.9964	0.9966	0.9964	0.9964	0.9962
WF	0.7380	0.5894	0.8024	0.9513	0.9208	0.8481	0.9987	0.9986	0.9985	0.9987	0.9961
GW	0.7705	0.6128	0.8370	0.8373	0.9363	0.8723	0.9988	0.9988	0.9986	0.9991	0.9978
GF1	0.8495	0.6000	0.9045	0.8303	0.9000	0.8239	0.9968	0.9986	0.9944	0.9824	0.9904
GF2	0.7576	0.5736	0.9193	0.9085	0.9077	0.8444	0.9985	0.9991	0.9979	0.9979	0.9945
GF3	0.8639	0.6972	0.9123	0.8927	0.8956	0.8824	0.9980	0.9993	0.9968	0.9922	0.9933
GWF1	0.8596	0.7679	0.8416	0.8661	0.8457	0.8043	0.9968	0.9990	0.9947	0.9838	0.9906
GWF2	0.8874	0.7108	0.9004	0.9087	0.8954	0.8279	0.9988	0.9994	0.9982	0.9981	0.9940
GWF3	0.8727	0.7510	0.8647	0.8806	0.8509	0.8957	0.9980	0.9993	0.9967	0.9925	0.9925

Methods	IQI						SSIM				
	Lena	Darkhair	Blonde	Peppers	Mandrill	Barbara	Lena	Darkhair	Blonde	Peppers	Mandrill

GF	0.5079	0.4683	0.5108	0.4718	0.7405	0.6825	0.8331	0.8793	0.8287	0.8393	0.9146
WF	0.5885	0.5816	0.6017	0.7470	0.7458	0.7019	0.8998	0.9190	0.8972	0.8622	0.7598
GW	0.6001	0.6107	0.6103	0.6683	0.8326	0.7048	0.9065	0.9281	0.9028	0.9139	0.9259
GF1	0.5412	0.5514	0.5973	0.5776	0.5439	0.4920	0.8898	0.9248	0.6942	0.6859	0.5551
GF2	0.5667	0.5912	0.6826	0.7031	0.6677	0.5919	0.8847	0.9213	0.7902	0.8174	0.6834
GF3	0.5900	0.6471	0.7137	0.7002	0.6406	0.5891	0.9108	0.9550	0.8094	0.8105	0.6527
GF4	0.6130	0.6482	0.7355	0.7429	0.6738	0.5900	0.9191	0.9710	0.8425	0.8605	0.6886
GWF1	0.5429	0.6177	0.6356	0.6357	0.4984	0.4949	0.8899	0.9458	0.7463	0.7510	0.5239
GWF2	0.6157	0.6448	0.7396	0.7558	0.6348	0.5821	0.9205	0.9440	0.8477	0.8720	0.6560
GWF3	0.5908	0.6520	0.6917	0.6972	0.5638	0.5890	0.9109	0.9530	0.8032	0.8171	0.5884
GWF4	0.6128	0.6488	0.7339	0.7486	0.6241	0.7503	0.9186	0.9463	0.8412	0.8624	0.6452

Methods	MSE						RMSE				
	Lena	Darkhair	Blonde	Peppers	Mandrill	Barbara	Lena	Darkhair	Blonde	Peppers	Mandrill

GF	131.76	106.06	150.66	132.06	141.35	131.60	11.48	10.30	12.27	11.49	11.89
WF	45.99	42.07	58.53	49.31	139.63	86.45	6.78	6.49	7.65	7.02	11.82
GW	44.57	36.35	57.37	34.60	79.17	90.04	6.68	6.03	7.57	5.88	8.90
GF1	113.90	46.09	226.43	627.27	347.98	351.54	10.67	6.79	15.05	25.05	18.65
GF2	52.21	29.51	83.38	76.40	198.78	217.05	7.23	5.43	9.13	8.74	14.10
GF3	71.33	21.59	128.04	279.79	242.21	248.31	8.45	4.65	11.32	16.73	15.56
GF4	47.88	21.75	84.00	112.34	198.04	248.41	6.92	4.78	9.17	10.60	14.07
GWF1	114.28	31.75	210.18	577.60	335.33	355.50	10.69	5.63	14.50	24.03	18.31
GWF2	43.69	20.26	73.73	69.65	213.93	221.41	6.61	4.50	8.59	8.35	14.63
GWF3	71.29	21.01	132.31	269.16	267.95	254.04	8.44	4.58	11.50	16.41	16.37
GWF4	48.03	19.74	84.27	110.90	222.33	106.03	6.93	4.44	9.18	10.53	14.91

Methods	ERR3						ERR4				
	Lena	Darkhair	Blonde	Peppers	Mandrill	Barbara	Lena	Darkhair	Blonde	Peppers	Mandrill

GF	13.30	12.73	13.93	13.41	13.48	13.50	14.80	14.72	15.24	14.96	14.77
WF	8.26	8.50	9.29	8.76	14.25	11.73	9.63	10.27	10.85	10.46	16.44
GW	8.09	7.83	9.22	7.25	10.84	11.94	9.41	9.41	10.77	8.52	12.66
GF1	16.09	9.24	21.85	41.96	23.19	25.72	21.78	12.03	28.88	57.94	27.39
GF2	8.98	6.88	12.12	12.22	17.57	20.67	10.87	8.19	15.65	16.89	20.89
GF3	12.07	6.23	16.67	29.40	19.31	21.77	15.97	8.04	22.41	42.65	22.76
GF4	9.18	6.34	12.72	16.29	17.52	21.96	11.60	8.02	16.46	22.78	20.78
GWF1	16.09	8.15	21.27	40.47	22.78	25.96	21.73	11.33	27.99	55.96	26.79
GWF2	8.61	5.73	11.82	12.05	18.14	20.89	10.78	6.88	15.33	16.67	21.36
GWF3	12.04	6.17	16.36	27.95	20.17	22.16	15.89	8.00	21.39	40.22	23.55
GWF4	9.22	5.68	12.71	16.03	18.42	13.44	11.67	6.88	16.40	22.15	21.60

**Table 3:** Comparison of performance parameters for different images with noise variance equal to 0.01.

Methods	LMSE						*β*			
	Lena	Darkhair	Blonde	Peppers	Mandrill	Barbara	Lena	Darkhair	Blonde	Peppers
GF	6.410	22.745	2.419	11.595	3.321	0.882	0.326	0.167	0.484	0.197
WF	1.255	5.895	0.615	0.287	0.654	0.384	0.412	0.141	0.603	0.783
GW	1.101	4.585	0.581	1.267	0.603	0.390	0.431	0.143	0.618	0.442
GF1	1.967	4.289	2.891	6.751	1.462	1.323	−0.150	0.012	−0.203	−0.526
GF2	1.059	2.076	0.910	0.478	0.929	0.983	0.330	0.121	0.358	0.626
GF3	1.137	1.664	1.545	3.038	1.074	1.033	0.067	0.083	−0.056	−0.269
GF4	0.833	1.754	0.894	0.766	0.925	1.056	0.411	0.092	0.364	0.487
GWF1	1.971	2.432	2.297	5.948	1.241	1.322	−0.153	−0.002	−0.285	−0.572
GWF2	0.794	1.178	0.763	0.408	0.881	0.998	0.455	0.202	0.489	0.713
GWF3	1.136	1.283	1.355	2.726	1.022	1.034	0.067	0.103	−0.068	−0.289
GWF4	0.837	1.061	0.896	0.775	0.900	0.906	0.406	0.214	0.369	0.487

Methods	SNR (dB)						PSNR (dB)			
	Lena	Darkhair	Blonde	Peppers	Mandrill	Barbara	Lena	Darkhair	Blonde	Peppers

GF	42.55	43.26	42.48	42.67	42.15	42.10	26.93	27.88	26.35	26.92
WF	51.97	52.56	50.86	54.30	47.18	45.40	31.64	32.53	30.54	32.74
GW	43.82	50.50	38.91	29.15	34.26	33.57	27.57	31.49	24.58	20.16
GF1	43.79	53.73	39.55	29.86	34.58	33.47	27.55	33.11	24.90	20.51
GF2	50.59	54.37	47.58	47.43	39.13	37.76	30.95	33.43	28.92	29.30
GF3	52.14	57.64	48.65	48.24	38.49	37.58	31.73	35.06	29.45	29.70
GF4	47.88	57.08	43.86	36.16	37.41	36.59	29.60	34.79	27.06	23.66
GWF1	47.89	57.32	43.57	36.50	36.53	36.39	29.60	34.91	26.91	23.83
GWF2	51.35	57.67	47.52	44.08	39.16	36.45	31.33	35.02	28.89	27.63
GWF3	51.32	57.86	47.49	44.20	38.15	43.87	31.32	35.18	28.87	27.68
GWF4	51.70	51.29	50.69	51.24	42.19	45.75	31.50	31.89	30.46	31.20

**Table 4:** Comparison of edge preserving parameters.

Reference	Method	FoM	IQI	SSIM	PSNR	*ρ*	MSE	SNR
[2, 31]	Kaun	0.8601	0.5425	0.9166	31.26	0.9986	48.60	51.22
[2, 20]	Lee	0.8738	0.5379	0.9140	31.14	0.9986	50.05	50.96
[2, 19]	Frost	0.8034	0.5970	0.9046	**31.64**	0.9987	44.53	51.98
[10]	DPAD	0.6290	0.5750	0.8721	29.61	0.9982	71.22	47.90
[2, 7]	SRAD	0.7763	0.5822	0.8834	25.70	0.9960	174.85	40.10
[2, 8]	PMAD	0.5916	0.4468	0.8396	27.04	0.9964	128.60	42.76
[9]	CED	0.6002	0.4896	0.8126	26.38	0.9958	149.61	41.45
[30]	hmedian	0.6668	0.5332	0.8662	29.60	0.9980	71.32	47.88
[25]	PPB	0.8655	**0.6411**	**0.9535**	**33.99**	**0.9993**	**25.95**	**56.67**
[22]	AFTV	0.5219	0.5095	0.8209	27.14	0.9965	125.60	42.97
[24]	NLM	**0.8881**	**0.6377**	**0.9403**	25.44	0.9971	185.76	39.57
[15]	GLM	0.8218	0.5846	**0.9309**	**32.15**	**0.9989**	39.62	**52.99**
[13]	ProbShrink	0.8377	0.5678	0.8970	29.01	0.9977	81.69	46.71
[14]	MPT	0.5045	0.5087	0.7726	26.80	0.9962	135.72	42.30
[26]	RNLA	0.7869	0.5125	0.8884	29.95	0.9981	65.72	48.59
[16]	OWT	0.6377	0.4714	0.8086	25.71	0.9951	174.59	40.11
[17]	MBR	0.7634	0.5560	0.9001	27.06	0.9964	127.85	42.81
[3]	PSBE	0.5281	0.4702	0.8070	25.67	0.9951	176.16	40.03
[32]	Curvelets	0.6211	0.4707	0.8068	25.66	0.9950	176.66	40.01
[23]	FBL	0.7804	0.5446	0.9087	30.54	0.9984	57.45	49.76
[21]	ATV	0.8477	0.5856	0.9309	**32.33**	**0.9989**	38.03	51.22
Proposed	GW	0.7705	**0.6001**	0.9065	27.57	**0.9988**	44.57	43.82
	GF3	**0.9099**	**0.6002**	**0.9228**	30.09	0.9982	63.66	**52.14**
	GWF1	0.8671	0.5431	0.8905	27.57	0.9968	113.81	47.89
	GWF2	**0.8819**	**0.6163**	**0.9202**	**31.73**	**0.9988**	**43.62**	51.35

SRAD: speckle reducing anisotropic diffusion, PMAD: Perona and Malik AD, CED: coherence enhancing diffusion, hmedian: hybrid median, AFTV: adaptive fidelity total variation, GLM: generalized likelihood method, ProbShrink: probability based shrinkage, MPT: multiscale product thresholding, RNLA: Ripplet with nonlinear approximation, OWT: orthogonal wavelet thresholding, MBR: m-band ridgelet, PSBE: posterior sampling based estimation, FBL: fast bilateral, ATV: anisotropic total variation.
